# Atrial Septal Defect, Pulmonary Arterial Hypertension, and Diastolic Left Heart Failure: When 3 Players Come into the Game

**DOI:** 10.1161/CIRCHEARTFAILURE.123.010545

**Published:** 2024-03-21

**Authors:** Tobias Rutz, John-David Aubert, Maurice Beghetti, Eric Eeckhout, Olivier Muller, Judith Bouchardy, Patrick Yerly

**Affiliations:** Service of Cardiology (T.R., E.E., O.M., J.B., P.Y.), Lausanne University Hospital and University of Lausanne, Switzerland.; Division of Pulmonology (J.-D.A.), Lausanne University Hospital and University of Lausanne, Switzerland.; Pediatric Cardiology Unit and Centre Universitaire Romand de Cardiologie et Chirurgie Cardiaque Pédiatrique, Children’s University Hospitals, Lausanne and Geneva, Switzerland (M.B.).

**Keywords:** atrial septal defect, congenital heart disease, diastolic heart failure, heart failure with preserved ejection fraction, pulmonary arterial hypertension, renin-angiotensin-system

Pulmonary arterial hypertension (PAH) usually precludes atrial septal defect (ASD) closure, but selected cases can still undergo intervention on PAH therapy. In addition to the risk of right ventricular (RV) failure, older patients may deteriorate postoperatively due to heart failure with preserved ejection fraction (HFpEF).

## CASE PRESENTATION

**Figure 1.**
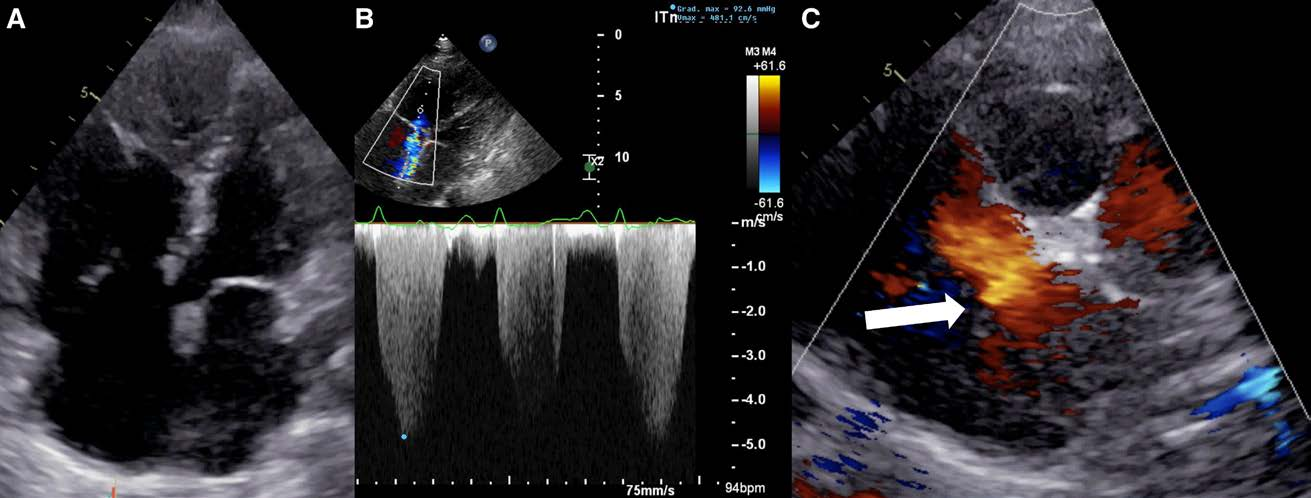
**Transthoracic echocardiographic findings at presentation in the tertiary center. A**, Transthoracic echocardiography at presentation showing right cavities dilatation and septal bulging into the left ventricle in apical 4-chamber view. **B**, Mild tricuspid regurgitation with peak tricuspid regurgitation velocity at 4.81 m/s (93-mm Hg gradient between the right ventricle and the right atrium according to the Bernoulli equation). **C**, Subcostal view of the so far unknown atrial left-to-right shunting (white arrow).

**Figure 2.**
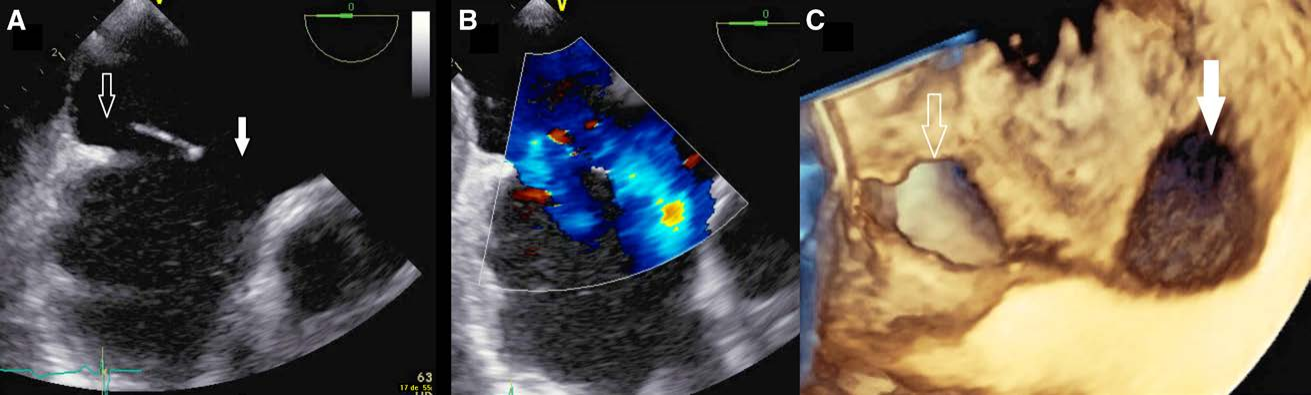
**Transesophageal echocardiography showing 2 atrial septal defects. A**, White arrow, secundum type atrial septal defect (ASD) in loco typico; white bordered arrow, ASD at the junction with the superior vena cava and the right upper pulmonary vein. **B**, ASDs with color Doppler showing left-to-right shunting. **C**, 3-dimensional reconstruction of both ASDs.

**Figure 3.**
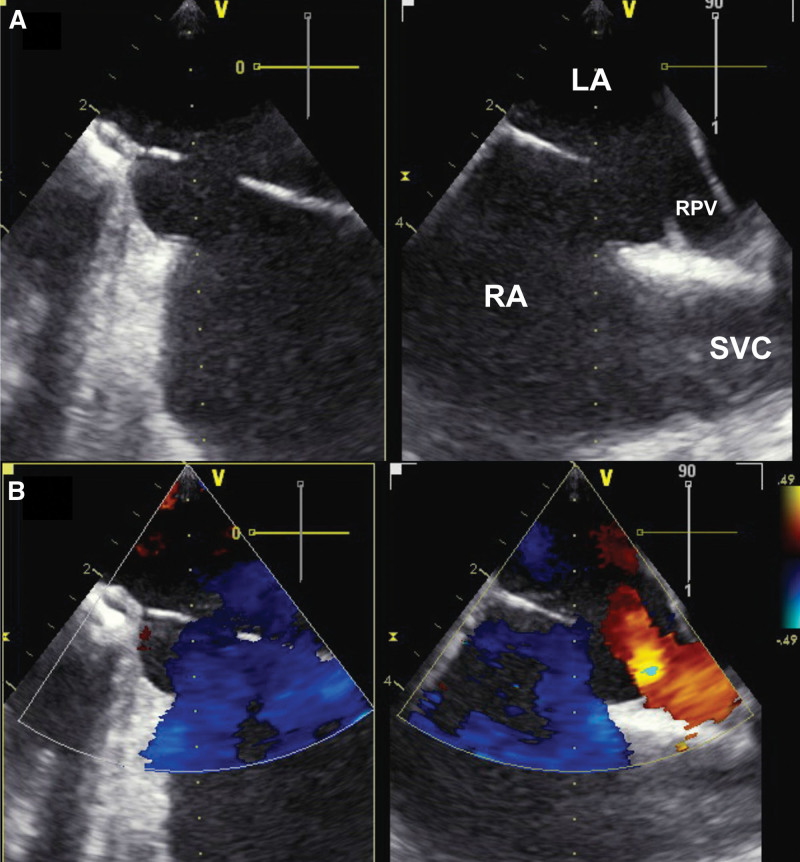
**Transesophageal echocardiography of the smaller atrial septal defect. A**, Biplane transesophageal echocardiography of the smaller atrial septal defect (ASD). It is located close to the arrival of the superior vena cava (SVC) and right upper pulmonary vein (RPV) precluding an interventional closure due to the risk of the obstruction of the SVC or RPV. **B**, Biplane transesophageal echocardiography of the smaller ASD with color Doppler. LA indicates left atrium; and RA, right atrium

A 48-year-old woman on sildenafil 20 mg tid and bosentan 125 mg bid for idiopathic PAH for 6 years was referred for clinical worsening. Sildenafil was increased to 40 mg tid with no improvement (Table). Transthoracic echocardiography revealed a hitherto unknown atrial left-to-right (L-R) shunt, right heart dilatation, moderate RV dysfunction, high RV to right atrial gradient, and normal left ventricular (LV) function (Figure 1A through 1C). Transesophageal echocardiography showed 2 ASDs, 1 (16×23 mm) secundum type, and 1 (12×15 mm) at the junction of the superior vena cava and right upper pulmonary vein (Figures 2 and 3; Videos S1 through S3). Cardiac magnetic resonance and right-left heart catheterization revealed high pulmonary vascular resistance (PVR) and mild L-R shunt (Table S1). The diagnosis was changed to PAH associated with congenital heart disease, and Selexipag was added and titrated to a maximal tolerated dose (400 µg bid).^1^

**Table.**
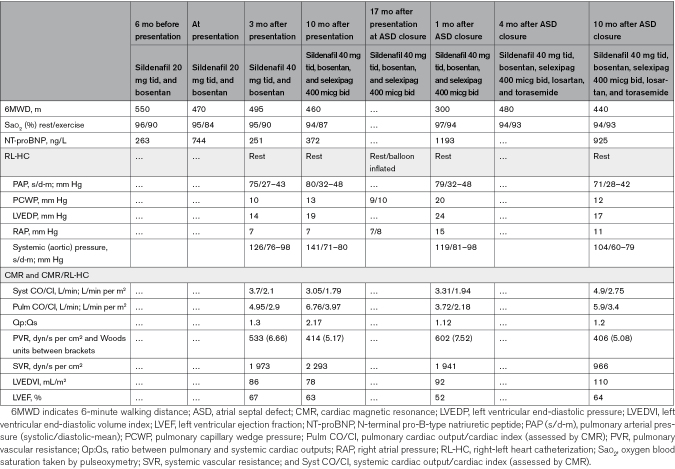
Clinical, Biologic, and Hemodynamic Characteristics of the Patients During Follow-Up

**Figure 4.**
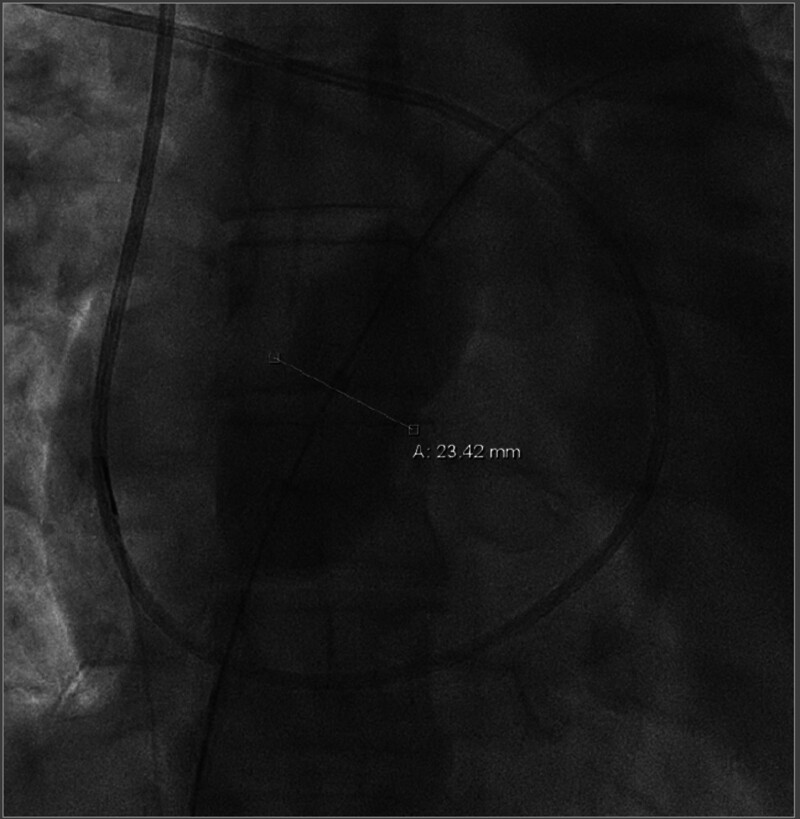
**Balloon test occlusion of atrial septal defect.** Balloon test occlusion with measurement of the size of the atrial septal defect in loco typico.

**Figure 5.**
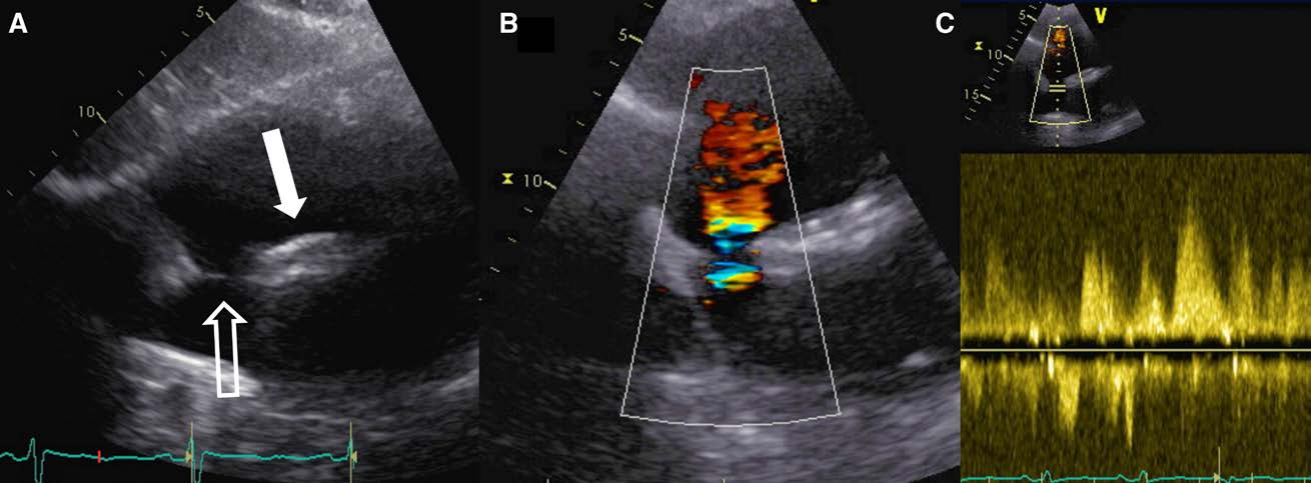
**Transthoracic echocardiography after partial closure of atrial septal defects. A**, Subcostal view showing the Amplatzer device (white arrow) and the persistent atrial septal defect (white bordered arrow) close to the arrival of the right upper pulmonary vein. **B**, Left-to-right shunting on color Doppler through the persistent atrial septal defect. **C**, Left-to-right shunting on pulse-waved Doppler through the persistent atrial septal defect.

After 4 months, the patient remained in New York Heart Association class III, whereas PVR decreased and L-R shunt increased (Table). Despite an elevated PVR and LV filling pressures (LV end-diastolic pressure, 19 mm Hg), the larger ASD was closed after balloon test occlusion with an Amplatzer 24-mm occluder (Figures 4 and 5; Table), while the smaller ASD was kept open as a pop-off valve.^1^

Four weeks later, the patient presented with worsening dyspnea and pulmonary congestion. Repeat right-left heart catheterization showed less L-R shunting, persistently low systemic cardiac output (sCO), and markedly increased systemic vascular resistance and LV filling pressures. After starting losartan (50 mg bid) and torasemide, the patient recovered to New York Heart Association class II. Right-left heart catheterization performed 9 months later confirmed normalized LV filling pressures and sCO (Table).

## DISCUSSION

Our case illustrates 3 difficult aspects of ASD-associated PAH management.

### ASD Diagnosis

The ASDs were not detected on transthoracic echocardiography before referral to our center, but no bubble study was performed. L-R shunting was possibly missed on color Doppler due to high RV filling pressures, thus reducing shunt flow. With PDE5i (phosphodiesterase-5 inhibitor) uptitration, PVR decreased and RVEF improved, shifting RV filling to the left of the diastolic pressure-volume curve. Consequently, the RV was more compliant than the LV, increasing the L-R shunt and facilitating shunt detection. To avoid missing ASDs, transthoracic echocardiography with agitated saline, transesophageal echocardiography, and cardiac magnetic resonance with shunt calculation should be routinely performed in apparently idiopathic PAH.

### Defect Closure

ASD closure is appealing in patients with PAH and persistent L-R shunting as it may improve pulmonary arterial pressure and functional class.^2^ If PVR is ≥5 Woods units, the closure could, however, be harmful because it would sacrifice the pop-off valve function of the ASD that would maintain sCO and limit organ congestion in the case of progressive PAH and severe RV failure.^3^ PAH drugs can be attempted, but if PVR remains borderline as in the present case, closure is theoretically a class III indication.^4^ Nevertheless, centers with high levels of expertise in the fields of PAH and congenital heart disease reported encouraging experiences with the implantation of fenestrated devices that keep the pop-off valve functioning.^5^ In our patient, a natural valve existed with the second ASD, thus facilitating the procedure. As PAH evolution remains difficult to predict after ASD closure, careful follow-up remains mandatory.

### Postinterventional HFpEF

LV filling pressures and the L-R shunt increased concomitantly under PAH treatment intensification. As the patient remained symptomatic, NT-proBNP (N-terminal pro-B-type natriuretic peptide) remained low, and the patient had a natural pop-off valve, the potential benefits of shunt restriction with improved symptoms were felt to be greater than the risks by the multidisciplinary team despite the patient having a hemodynamic diagnosis of HFpEF with an LV end-diastolic pressure of 19 mm Hg before closure.^6^ Indeed, HFpEF complicating ASD closure in older patients can usually be overcome with the use of fenestrated devices.^7^ Nevertheless, our patient’s remaining ASD was obviously too small to avoid postoperative overt HFpEF, which we treated with loop diuretics and losartan.

While diuretics helped to lower filling pressures, they certainly did not contribute to increasing sCO. Septal interference can neither be explanatory as it usually resolves within 30 days after intervention.^8^ Considering that the persistently low postprocedural sCO may account for a major trigger for renin release, we speculated that excessive renin-angiotensin-aldosterone system (RAAS) stimulation and increased systemic vascular resistance were involved in preventing adequate LV emptying.^9^ The combination of diuretics and RAAS inhibition could, therefore, have improved filling pressures and sCO, which translated into improved New York Heart Association class and functional capacity. We acknowledge that our considerations are in contrast with the results of major trials that failed to show any significant efficacy of RAAS inhibition in HFpEF, but they are still consistent with recent data showing improved outcomes with RAAS inhibition in a subset of patients with HFpEF with a phenotype of RAAS activation.^10^

This case highlights 3 essential challenges in PAH-associated ASD and suggests a potential role for RAAS inhibition in ASD-related HFpEF.

## ARTICLE INFORMATION

### Sources of Funding

None.

### Disclosures

None.

### Supplemental Material

Table S1

Videos S1–S3
